# Role of three-dimensional Doppler ultrasonography and leukemia inhibitory factor from endometrial secretion in predicting endometrial receptivity in IVF treatment: a pilot study

**DOI:** 10.1007/s00404-022-06450-2

**Published:** 2022-02-28

**Authors:** Ivan Sini, Nining Handayani, Alida Harahap, Arief Boediono, Budi Wiweko, Wachyu Hadisaputra, Soegiharto Soebijanto, Tri Aprilliana, Arie A. Polim, Aryando Pradana

**Affiliations:** 1 Morula IVF Jakarta Clinic, Jl. Teuku Cik Ditiro 12A Menteng, Jakarta, Indonesia; 2 IRSI Research and Training Centre, Jakarta, Indonesia; 3 Doctoral Program, Faculty of Medicine, University of Indonesia, Jakarta, Indonesia; 4 Eijkman Institute for Molecular Biology, Ministry of Research and Technology/National Agency for Research and Innovation, Jakarta, Indonesia; 5 Department of Anatomy, Physiology and Pharmacology, IPB University, Bogor, Indonesia; 6 Division of Reproductive Endocrinology and Infertility, Department of Obstetrics and Gynecology, Faculty of Medicine, University of Indonesia, Jakarta, Indonesia; 7 Department of Obstetrics and Gynecology, School of Medicine and Health Sciences, Atmajaya Catholic University of Indonesia, Jakarta, Indonesia

**Keywords:** Endometrial receptivity, Leukemia Inhibitory factor, Power Doppler ultrasound, Sub-endometrial vascularization

## Abstract

**Purpose:**

This pilot study aimed to evaluate the potential synergistic role of three-dimensional power Doppler angiography ultrasound and the expression of Leukemia Inhibitory Factor (LIF) protein in predicting the endometrial receptivity of fresh In-Vitro Fertilization (IVF) cycles.

**Materials and methods:**

This prognostic cohort study involved 29 good prognosis women who underwent fresh IVF cycles with fresh blastocysts transfer. Serial measurements of sub-endometrial parameters including vascularity index (VI), flow index (FI), and vascularization flow index (VFI) were conducted consecutively via power Doppler angiography on the day of oocyte maturation trigger, oocyte retrieval, and blastocyst transfer. Aspiration of endometrial secretion was performed on the day of embryo transfer.

**Results:**

The mean index of VI and VFI on the trigger and oocyte retrieval day and also LIF protein concentration at the window of implantation were significantly higher in clinically pregnant women than that of the non-pregnant women (*p* < 0.05). The area under the curve (AUC) of VI and VFI was shown to have a powerful predictive value to forecast receptive endometrium on either trigger day (0.788 and 0.813, respectively) or oocyte retrieval day (0.813 and 0.818). Likewise, LIF concentration on the day of embryo transfer was adequate to become a predictor for endometrial receptivity (AUC 0.874). A combination of the VI and VFI on the trigger day and LIF concentration at specific cut-off values (VI > 5.381, VFI > 1.483, LIF 703.5 pg/mL) produced an algorithm with high AUC (0.881) and high specificity (94.4%) for an adequate prediction of non-receptive endometrium.

**Conclusion:**

VI and VFI index assessed on maturation trigger day and the expression of LIF protein concentration at the window of implantation provided sufficient information to predict endometrial receptivity. A large randomized control trial is needed to validate these findings.

## Introduction

Successful implantation reflects an excellent bi-directional communication and interaction between embryos and maternal endometrium at the receptive phase through a molecular pathway. Endometrial receptivity which is reflected by clinical pregnancy is defined as endometrium with high implantation potential characterized by histological changes that are driven by consecutive actions of steroid hormones, estradiol, and progesterone, in the mid-luteal phase [1]. Despite various clinical and laboratory improvements in *In-Vitro* Fertilization (IVF) treatment for infertile couples, the search for markers in predicting endometrial receptivity remains inconclusive. Investigation on several potential markers including multi-omics of endometrium (i.e., genomics, proteomics, secretomes, metabolomics), cytokines profile, and ultrasound Doppler signals have long been explored, but none of them have yielded a gold standard diagnostic test [2].

One of non-invasive predictors of endometrial receptivity in IVF is through ultrasound assessment of endometrial and sub-endometrial vascularization. Adequate endometrial blood flow and perfusion are prerequisites of endometrial preparation for embryo implantation and is essential to endometrial receptivity. Kupesic and Coworkers indicated that lack of sub-endometrial perfusion measured with 3D power Doppler during peri-ovulation was associated with implantation failure in IVF cycles [3]. Nonetheless, despite the promising predictive value of this marker in various studies [4, 5], correlating it with the implantation potential remains controversial [2, 6, 7].

Leukemia inhibitory factor (LIF) is a well-recognized endometrial cytokine that possesses an important role in the event of implantation [8, 9]. LIF is a pleiotropic factor of the interleukin-6 family which is prominently transcribed and expressed, during the early secretory phase in both the luminal and glandular epithelium [8]. Its interactions with other proteins and factors are essential to promote trophoblastic cell motility.

In humans, altered expression of LIF in infertile women with unexplained infertility has been demonstrated [10]. Other studies also found that women with recurrent implantation failure might have an initial dysregulation of the immune and inflammatory response of LIF, progestagen-associated endometrial protein (PAEP), and interleukin-6 signal transducer (IL6ST) [11]. In concordance with those findings, blastocyst has been shown to express LIF receptor (LIFR) [12], providing evidence for the important regulatory function of LIF during the implantation process. Previous investigations of LIF in relation to implantation have been focused mainly on the population study of infertile women versus fertile women [10, 11, 13], while the measurement of LIF in endometrial cavity during peri-implantation of a fresh IVF cycle has yet been studied.

The objective of this preliminary study is to evaluate the combined use of three-dimensional Doppler ultrasound of the sub-endometrial vascularity indices during maturation trigger, oocyte retrieval, embryo transfer as a clinical marker and LIF concentration at the implantation window as a biological marker to predict endometrial receptivity in a fresh IVF cycle.

## Material and method

### Patients

The subjects recruited were infertile patients who underwent their first IVF cycle at Morula IVF Clinic Jakarta, Indonesia between January 2019—June 2020. The patients were counseled to participate in the study at the beginning of the stimulation treatment. The inclusion criteria included regular menstrual cycle, no uterine abnormality, and normal ovarian reserve. The exclusion criteria were women with uterine pathologies (i.e., uterine myoma, endometrial polyp, endometriosis, and adenomyosis) and those with a history of uterine surgery (i.e., myomectomy, adenomyosis resection, and curette operation of more than three times). Receptive endometrium is reflected by clinical pregnancy.

### Assisted reproductive treatment

All patients were subjected to ovarian stimulation with antagonist protocol. Gonadotropin recombinant FSH (rFSH, Gonal F^®^, Merck Serono) or a combination of recombinant FSH and recombinant LH (rFSH/rLH, Pergoveris^®^, Merck Serono) or urinary Human Menopausal Gonadotropin (uHMG) were administered daily starting on day 2 or 3 of the menstrual cycle. The initial dose ranged between 150 to 300 IU per day. Injection of 0.25 mg GnRH antagonists (Cetrotide^®^, Merck KGaA) was commenced on day 5 of the stimulation. Recombinant Human Chorionic Gonadotropin (rHCG 250 mcg, equivalent to 6,500 IU, Ovidrel^®^, Merck Serono) was given as the final oocyte maturation injection when three dominant follicles had reached 18 mm in diameter. Oocyte retrieval procedure was performed 36 h later under mild sedation. Intracytoplasmic Sperm Injection (ICSI) was performed in sperm factor infertility and teratozoospermia cases. All embryos were cultured to blastocyst stage. Good quality blastocysts were evaluated on day 5 after ICSI procedure according to the Gardner criteria (grade 3–5 of blastocoel expansion with a combined score of inner cells mass and trophoblast cells of AA, AB, or BA) [14]. Embryo transfer procedures with maximum two good blastocysts were performed with a soft catheter (K-JET-7019-ET, Cook, USA). All patients were given progesterone for luteal support starting on retrieval day until confirmation of pregnancy (vaginal progesterone: 200 mg Utrogestan^®^ (Besins Healthcare, Belgium)/12 h and 90 mg Crinone^®^ (Merck, Serono)/day). Positive Beta HCG result was confirmed with transvaginal ultrasound 4 weeks after OPU.

### Three dimensional Doppler ultrasound measurement

Transvaginal ultrasound scans were utilized to evaluate follicular and endometrial development. Three dimensional Doppler assessments were performed during the day of maturation trigger, oocyte retrieval, and embryo transfer. Histogram data were captured with built-in VOCAL II (Virtual Organ Computer-aided Analysis) software in all ultrasound machines used for this study (Voluson S10, General Electric, USA). The power Doppler settings were adjusted as follows: color gain 45.6; pulse repetition frequency, 1.0; color power, 3; with 145° View. The uterus was visualized in a longitudinal view. In power Doppler mode, scanning is performed at every 9° rotation which resulted in 20 layer of endometrium slices. The sub-endometrium region was considered as 5-mm shell outside of endometrial contour as it is of most relevance to represent radial and spiral vessels [15]. The measurement was taken in the sagittal plane and indices of VI, FI, and VFI were calculated automatically. Three well-trained clinicians were involved to capture the data with 0.771 of interclass coefficient correlation. VI represents the sub-endometrial vascularity or density of vessels. FI expresses the mean of blood flow intensity, while VFI reflects the endometrial vascularity and perfusion [15].

### Endometrial secretion collection

Endometrial secretion was collected immediately before blastocyst transfer. The procedure was performed as described by previous studies [16, 17]. As the patient was lying in a lithotomy position prior to embryo transfer, a speculum was inserted and the cervix was cleaned with saline. The sterile soft catheter was inserted into the uterine cavity under ultrasound guidance. As the catheter tip reached the mid cavity, the endometrial secretion sample was aspirated with a 10 ml syringe. All samples were then stored at − 20 °C.

### LIF analysis

Endometrial secretion samples were diluted with Phosphate Buffered Saline (PBS) solution up to a volume of 100 µL. Human LIF enzyme-linked immunosorbent assay kit (ELISA) (Invitrogen/Thermo Fischer Scientific, US) was used according to the manufacturer’s protocol to analyze the samples. Sensitivity of the ELISA kit was in the range of < 5–500 pg/mL. Dilution factor was calculated for each sample and was used to correct the calculated LIF concentration (pg/mL).

### Statistical analysis

Statistical analyses were performed using the Statistics Package for Social Sciences (SPSS) version 20.0 (SPSS Inc, Chicago, IL, USA). All variables were evaluated for normal data distribution with the Shapiro–Wilk test. SQRT method was utilized to normalize non-normal data distribution. Numerical variable analysis was performed using T-test or Mann–Whitney test. Categorical variables were analyzed using Chi-squared tests. Multiple analysis using generalized linear model (GLM) method was used to adjust potential confounders. The probability of < 0.05 was considered statistically significant.

## Results

Women participated in this study were relatively young with normal BMI. Most indications for IVF were primary infertility with sperm factor, unexplained infertility and recurrent IUI failures. Clinical characteristics of the study subjects were in good prognosis group and responded accordingly (Table 1). All women underwent embryo transfer with good quality blastocyst(s). The pregnancy rate was 37.9% with 11 achieved pregnancy, while the remaining subjects (*n* = 18) did not get pregnant. Endometrial secretion was successfully obtained from all subjects with median volume of 3 µL (min–max: 1–10 µL).

**Table 1:** Baseline, clinical characteristics, and embryology results of studied subjects

Parameters	Value (*n* = 29)
Baseline	
Female age (years)	31 (27–40)
Body mass index (Kg/m^2^)	23.83 ± 3.86
Type of infertility	
Primary infertility	24 (82.8%)
Secondary infertility	5 (17.2%)
Infertility etiologies	
Tubal factors	4 (13.8%)
Endometriosis	2 (6.9)
Sperm factors	12 (41.4%)
Unexplained infertility	11 (37.9%)
Recurrent IUI failure	7 (24.1%)
Clinical characteristics	
Anti Müllerian hormones (ng/mL)	2.94 (0.13–6.70)
Antral follicle count	13.45 ± 3.53
Basal follicle-stimulating hormone (mIU/mL)	7.15 ± 1.60
Basal luteinizing hormone (mIU/mL)	6 (0.1–15.1)
Basal estradiol (pg/mL)	36.50 ± 9.32
Basal progesterone (ng/mL)	0.14 (0.05–5.97)
Total gonadotropin usage (IU)	2.025 (1.050–3.000)
Estradiol on the trigger day (pg/mL)	2.212 (1.169–5.855)
Progesterone on the trigger day (ng/mL)	0.55 (0.20–1.35)
Endometrial thickness (mm)	12.09 ± 2.01
Embryology results	
Number of mature oocytes	7.97 ± 3.32
Number of top-quality blastocysts	2 (0–7)
Number of embryo transfers	1 (1–2)

Bivariate analysis showed statistically significant difference in the VI and VFI values on the maturation trigger day (*p* value < 0.05) between pregnant and non-pregnant women. The same results were also observed on the day of oocyte retrieval in which the VI and VFI values of pregnant women were significantly higher compared to the non-pregnant group (*p *value < 0.05). No significant difference was observed in the overall endometrial vascularization parameters assessed on the day of embryo transfer. Furthermore, the expression of LIF protein was significantly lower in non-pregnant women (*p* value = 0.003) (Table 2).

**Table 2:** Bivariate analysis of three-dimensional power Doppler angiography ultrasound and LIF protein concentration

Day of assessment	Parameters	Pregnant women (*n* = 11)	Non-pregnant women (*n* = 18)	*p* value
Maturation trigger	VI	8 ± 3.8	4.7 ± 5.9	0.020
	FI	28.9 ± 1.8	27.6 ± 3.6	NS
	VFI	2.4 ± 1.2	1.4 ± 1.9	0.020
Oocyte retrieval	VI	4.1 ± 3.1	1.9 ± 2.2	0.028
	FI	29.4 ± 4.6	28 ± 5.5	NS
	VFI	1.3 ± 1.1	0.6 ± 0.8	0.038
Embryo transfer	VI	3 ± 1.8	2.3 ± 2.5	NS
	FI	29.6 ± 4.2	28.3 ± 4.8	NS
	VFI	1 ± 0.7	0.8 ± 0.9	NS
Embryo transfer	LIF (pg/mL)	1200 ± 376	643 ± 636	0.003

Data were presented as mean and standard deviation

*NS* not significant. leukemia inhibitory factor; *VI* vascularization Index; *FI* flow index; vascularization flow index (VFI)

GLM analysis was performed to control the confounding factors which include female age, antral follicle count, basal LH, number of mature oocytes, and endometrial thickness. After confounder adjustments, significant differences persist in VI and VFI measurement during maturation trigger day and oocyte retrieval day and LIF protein concentration between pregnant and non-pregnant women.

Receiver operating curve (ROC) analyses were performed and presented in Table 3. The area under the curve of each parameter has good endometrial receptivity predictive value. Optimal cut-off points of VI and VFI index variable were > 5.381 and > 1.483, respectively (*p* value 0.003) on the maturation trigger day and > 2.048 and > 0.6335, respectively (*p* value 0.018) on the day of oocyte retrieval. LIF protein concentration with cut-off point of > 703.5 pg/mL (*p* value < 0.001) also offered good predictive value.

**Table 3:** ROC analysis of VI, VFI and LIF protein

Parameters	Sensitivity (%)	Specificity (%)	AUC	Standard error	*p* value	95% CI
VI maturation trigger	82	78	0.788	0.089	0.003	0.613–0.963
VI oocyte retrieval	73	78	0.813	0.094	0.018	0.628–0.998
VFI maturation trigger	82	78	0.813	0.089	0.003	0.638–0.988
VFI oocyte retrieval	72	78	0.818	0.098	0.018	0.627–1.000
LIF protein	100	78	0.874	0.073	< 0.001	0.731–1.000

*AUC* area under the curve; *VI* vascularization index; *VFI* vascularization flow index; *LIF* leukemia inhibitory factor

Several algorithms were constructed to formulate a combination of markers that predict endometrial receptivity by utilizing the cut-off values of VI and VFI on the day of maturation trigger and oocyte retrieval and the expression of LIF protein. Eventually, we found that the combination of VI and VFI on the day of maturation trigger with LIF concentration yielded better algorithm to predict non-receptive endometrium (AUC 0.881, sensitivity 81.8%, specificity 94.44%) as presented in Table 4.

**Table 4:** Algorithm for the classification of endometrial receptivity by combining the cut-off value of VI, VFI on the HCG day and LIF

Classification	LIF protein in the implantation window (pg/mL)	HCG day		*N* (%)
		VI	VFI	
Pregnant	> 703.5	> 5.381	> 1.483	10 (34.5)
Non-pregnant	> 703.5	< 5.381	< 1.483	19 (65.5)
	> 703.5	< 5.381	> 1.483	
	< 703.5	> 5,381	> 1.483	
	< 703.5	> 5.381	< 1.483	
	< 703.5	< 5.381	< 1.483	

*LIF* leukemia inhibitory factor; *VI* vascularization index; *VFI* Vascularization flow index; *HCG* human chorionic gonadotropin

## Discussion

The present preliminary study has indicated predictive potential of sub-endometrial VI, VFI index measurement on the day of maturation trigger and oocyte retrieval. Likewise the LIF protein concentration in endometrial secretion collected on the day of embryo transfer. Our study showed that sub-endometrial 3D power Doppler indices assessed on the day of embryo transfer were less effective in predicting the endometrial receptivity. In concordance with our results, a recent meta-analysis failed to show the significance of the aforemention parameters measured on the day of embryo transfer in differentiating women who achieved clinical pregnancy and women who did not [2].

A previous study observed that the VFI index on the maturation trigger day was highly predictive of endometrium receptive rather than the VI and FI indices [15]. Ng and Colleagues failed to signify all sub-endometrial vascularity indices to predict endometrial receptivity on either oocyte retrieval day [6] or the day of maturation trigger and embryo transfer [7]. These conflicting results corresponded to our findings which suggested further insights were to be evaluated to understand the correlation between endometrial vascularity indices as clinical markers for endometrial receptivity.

To the best of our knowledge, we are the first to provide preliminary data regarding the favorable endrometrial receptivity predictive value of LIF in fresh IVF cycle. The results of our study have supported the consistent claims of LIF as an important cytokine that regulates embryo implantation [8, 10, 11, 18]. Glandular and luminal epithelial cells of the endometrium and the blastocyst are known to express LIF and LIF receptors (LIF-R and gp130). Moreover, LIF expression in endometrium increases after ovulation day and attains its optimum concentration at the implantation window, suggesting the indispensable role of LIF and its receptors in the event of implantation. Endometrial LIF expression level could be measured by utilizing different sampling methods including uterine secretion aspiration [17], flushing [10, 19], or biopsy [10, 20]. While flushing and biopsy procedures might compromise the endometrial environment, secretion aspiration is safer and practicable as proven in this preliminary study wherein the endometrial secretions from all study subjects could be retrieved.

This study observed low VI and VFI indeces on maturation trigger and oocyte retrieval day was also followed by low expression of LIF on the day of embryo transfer. Junovich and colleagues [21] demonstrated the concordant decreased levels of total number endometrial NK cells and VI indexes in stimulated cycle. As NK cell was the prominent source of LIF production, that finding was congruent with our results therefore could suggest that an impaired endometrial NK cells homing had caused the altered expression of LIF on the day of embryo transfer.

Although VI and VFI on oocyte retrieval day and LIF level in endometrial secretion are likely to have good predictive values, the same combination of both vascularity indices and LIF biomarker measured on maturation trigger day also generated superior algorithm to predict non-receptive endometrium (specificity 94.4%). Such derived-algorithm can be practically used to assist the clinician in considering a selective freezing and defering the embryo transfer procedure especially when the vascularity indices and LIF expression were below the cut-off value.

The strength of our study is in the presented preliminary results which suggest the availability of a reliable, quick, and simple alternative method to measure endometrial receptivity by simply aspirating the uterine secretion sample at the implantation window for clinical practice in the IVF program. We supported the results of previous studies that a small amount of endometrial secretion could be gently aspirated for the analysis of the various implantation biomarkers without reducing the chance of pregnancy [16, 17]. This current study has confirmed the safety and effectiveness of the endometrial secretion aspiration method for pregnancy prediction. This preliminary study also unveils the potential clinical use of LIF as biological marker to predict endometrial receptivity. As compared to other endometrial receptivity assay or immunological testing, LIF expression in endometrial secretion can immediately be used in fresh IVF cycles as it is of most relevant to represent the environment of endometrium.

Nonetheless, this study also pertains to a limitation of a relatively small study population. Since this study only recruited infertile woman with good prognosis, a larger randomized clinical trial research should be performed to establish if the findings in this study are valid and could respresent the overall infertile population. Interclass coefficient correlation of this study was only 0.77 between the three well-trained IVF clinician suggesting potential deviation in the measurement of the USG indices. Furthermore, this study has yet to discover the in-depth association between the VI and VFI index and the expression of LIF during the implantation window. Notably, a concordant decreased of both VI and VFI index and low expression of LIF were more apparent in the non-pregnant group compared to the pregnant group. A further attempt to explore the relationship between vascularity indices and LIF expression during the implantation window is worth pursuing in the interest of gaining a broader perspective on the pathophysiology of aberrant LIF secretion that leads to impaired implantation.

In conclusion, our study provides preliminary evidence that measuring the sub-endometrial VI and VFI index on the day of maturation trigger could be useful in predicting endometrial receptivity. Endometrial LIF expression at the window of implantation is also a potential predictor for receptive endometrium in IVF. Moreover, a combination of the cut-off values of VI, VFI and LIF yielded a simple noteworthy algorithm for the prediction of non-receptive endometrium.

## Data Availability

Data is available upon reasonable request.

## References

[1] Lessey BA, Young SL (2019). What exactly is endometrial receptivity?. Fertil Steril [Internet].

[2] Craciunas L, Gallos I, Chu J, Bourne T, Quenby S, Brosens JJ (2019). Conventional and modern markers of endometrial receptivity: a systematic review and meta-analysis. Hum Reprod Update.

[3] Kupesic S, Kurjak A (1993). Uterine and ovarian perfusion during the periovulatory period assessed by transvaginal color Doppler. Fertil Steril.

[4] Elsokkary M, Eldin AB, Abdelhafez M, Rateb A, Samy M, Eldorf A (2019). The reproducibility of the novel utilization of five-dimensional ultrasound and power Doppler in the prediction of endometrial receptivity in intracytoplasmic sperm-injected women: a pilot prospective clinical study. Arch Gynecol Obstet.

[5] Mercé LT, Barco MJ, Bau S, Troyano J (2008). Are endometrial parameters by three-dimensional ultrasound and power Doppler angiography related to in vitro fertilization/embryo transfer outcome?. Fertil Steril.

[6] Ng EHY, Chan CCW, Tang OS, Yeung WSB, Ho PC (2006). The role of endometrial and subendometrial blood flows measured by three-dimensional power Doppler ultrasound in the prediction of pregnancy during IVF treatment. Hum Reprod.

[7] Ng EHY, Chan CCW, Tang OS, Yeung WSB, Ho PC (2009). Changes in endometrial and subendometrial blood flow in IVF. Reprod Biomed Online.

[8] Kondera-Anasz Z, Sikora J, Mielczarek-Palacz A (2004). Leukemia inhibitory factor: an important regulator of endometrial function. Am J Reprod Immunol.

[9] Singh M, Chaudhry P, Asselin E (2011). Bridging endometrial receptivity and implantation: network of hormones, cytokines, and growth factors. J Endocrinol.

[10] Tawfeek MA, Eid MA, Hasan AM, Mostafa M, El-Serogy HA (2012). Assessment of leukemia inhibitory factor and glycoprotein 130 expression in endometrium and uterine flushing: a possible diagnostic tool for impaired fertility. BMC Womens Health.

[11] Pathare ADS, Zaveri K, Hinduja I (2017). Downregulation of genes related to immune and inflammatory response in IVF implantation failure cases under controlled ovarian stimulation. Am J Reprod Immunol.

[12] Charnock-Jones DS, Sharkey AM, Fenwick P, Smith SK (1994). Endometrium at the time of implantation and the blastocyst contains. J Reprod Fertil.

[13] Laird SM, Tuckerman EM, Dalton CF, Dunphy BC, Li TC, Zhang X (1997). The production of leukaemia inhibitory factor by human endometrium: presence in uterine flushings and production by cells in culture. Hum Reprod.

[14] Gardner DK, Lane M, Stevens J, Schlenker T, Schoolcraft WB (2000). Blastocyst score affects implantation and pregnancy outcome: towards a single blastocyst transfer. Fertil Steril.

[15] Wu HM, Chiang CH, Huang HY, Chao AS, Wang HS, Soong YK (2003). Detection of the subendometrial vascularization flow index by three-dimensional ultrasound may be useful for predicting the pregnancy rate for patients undergoing in vitro fertilization-embryo transfer. Fertil Steril.

[16] van der Gaast MH, Beier-Hellwig K, Fauser BCJM, Beier HM, Macklon NS (2003). Endometrial secretion aspiration prior to embryo transfer does not reduce implantation rates. Reprod Biomed Online.

[17] Boomsma CM, Kavelaars A, Eijkemans MJC, Amarouchi K, Teklenburg G, Gutknecht D (2009). Cytokine profiling in endometrial secretions: a non-invasive window on endometrial receptivity. Reprod Biomed Online.

[18] Ledee-Ba-taille N, Lapree-Delage G, Taupin JL, Dubanchet S, Frydman R, Chaouat G (2002). Concentration of leukemia inhibitory factor (LIF) in uterine flushing fluid is highly predictive of embryo implantation. Hum Rep.

[19] Mikolajczyk M, Wirstlein P, Skrzypczak J (2007). The impact of leukemia inhibitory factor in uterine flushing on the reproductive potential of infertile women—a prospective study. Am J Reprod Immunol.

[20] Hasegawa E, Ito H, Hasegawa F, Hatano K, Kazuka M, Usuda S (2012). Expression of leukemia inhibitory factor in the endometrium in abnormal uterine cavities during the implantation window. Fertil Steril.

[21] Junovich G, Mayer Y, Azpiroz A, Daher S, Iglesias A, Zylverstein C (2011). Ovarian stimulation affects the levels of regulatory endometrial NK cells and angiogenic cytokine VEGF. Am J Reprod Immunol.

